# Whole-Body Vibration Therapy as a Modality for Treatment of Senile and Postmenopausal Osteoporosis: A Review Article

**DOI:** 10.7759/cureus.33690

**Published:** 2023-01-12

**Authors:** Anushree Singh, Anuj R Varma

**Affiliations:** 1 Medicine, Jawaharlal Nehru Medical College, Datta Meghe Institute of Medical Sciences, Wardha, IND

**Keywords:** dexa, bone mass density, postmenopausal osteoporosis, senile osteoporosis, whole body vibration therapy

## Abstract

Whole-body vibration therapy is an intentional biomechanical stimulation of the body using various frequencies of vibrations with the motive of health improvement. Ever since its discovery, this therapy has been extensively used in physiotherapeutic measures and the sports industry. For its property of increasing bone mass and density, space agencies use this therapy on astronauts who return to Earth after long-term space missions to regain lost bone and muscle mass. The potential of this therapy to restore bone mass encouraged researchers to look for its scope in the treatment of age-related bone degenerative diseases such as osteoporosis and sarcopenia, as well as in the correction of posture control and gait in geriatrics and post-menopausal women.

Osteoporosis and osteopenia account for roughly half of all fractures worldwide. These degenerative diseases also cause gait and posture changes. Bisphosphonates, monoclonal antibodies, parathyroid hormone fragments, hormone replacement therapies, and calcium and vitamin D supplementation are among the medical treatments available. Lifestyle changes and physical exercise are advised. However, vibration therapy’s scope as a treatment option is yet to be explored. The safe range of frequency, amplitude, duration, and intensity of the therapy is still to be determined.

This article is a review of the results of various clinical trials done in the last 10 years that target the effect of vibration therapy in both osteoporotic women and the elderly for the treatment of such ailments and deformities. We collected data from PubMed using advanced search and applied the exclusion criteria. In total, we analyzed nine clinical trials.

## Introduction and background

Fast-paced mechanical oscillations or vibrations of any variable frequency that are conducted to the body are called whole-body vibrations. We are exposed to oscillations throughout our daily routine, such as while driving a car, traveling on a train or plane, or even while using a smartphone [1]. Certain occupations are associated with exposure, like construction, agriculture, and the aviation industries. The human body is sensitive to vibrations of 1 Hz up to 100 Hz [2]. Vibrations below this range produce vestibular nausea and motion sickness, whereas a frequency higher than this range irritates and injures the musculoskeletal system [1]. It manifests as body pain and bone degeneration with prolonged exposure. For occupational safety, a frequency of 50 Hz was established as the standard [1,2].

Vibration training, vibrotherapy, biomechanical stimulation, or mechano-stimulation is the intentional exposure of vibrations of various frequencies and wavelengths in fixed joint positions for a definite duration. It can be pivotal, oscillating, or linear. It can be delivered through multiple devices, like a vibrating platform and belts. The plates vibrate at 90 Hz with brief oscillations, inducing acceleration equal to one-third of Earth's gravity [3]. Since its discovery, vibration therapy has been actively researched for its application in the medical field.

Old age is a difficult phase of life as the body slowly loses its integrity and strength. Slowly, numerous degenerative pathways get activated in the body after struggling with regular wear and tear over a very long time. Among all the degenerative diseases, senile osteoporosis, Alzheimer's disease, Parkinson's disease, and dementia have the highest incidence and are the most troublesome. Worldwide, annually, about 8.9 million fractures occur, most of which are due to osteopenia and osteoporosis [4]. Statistics predict that the prevalence of age-related osteoporotic fractures will rise by two- to threefold worldwide [4]. Osteoporotic fractures are predominantly seen in the arm, forearm, hip, and spine [4]. Out of these fractures, hip fractures are the most severe [4].

## Review

Methodology

**Figure 1:**
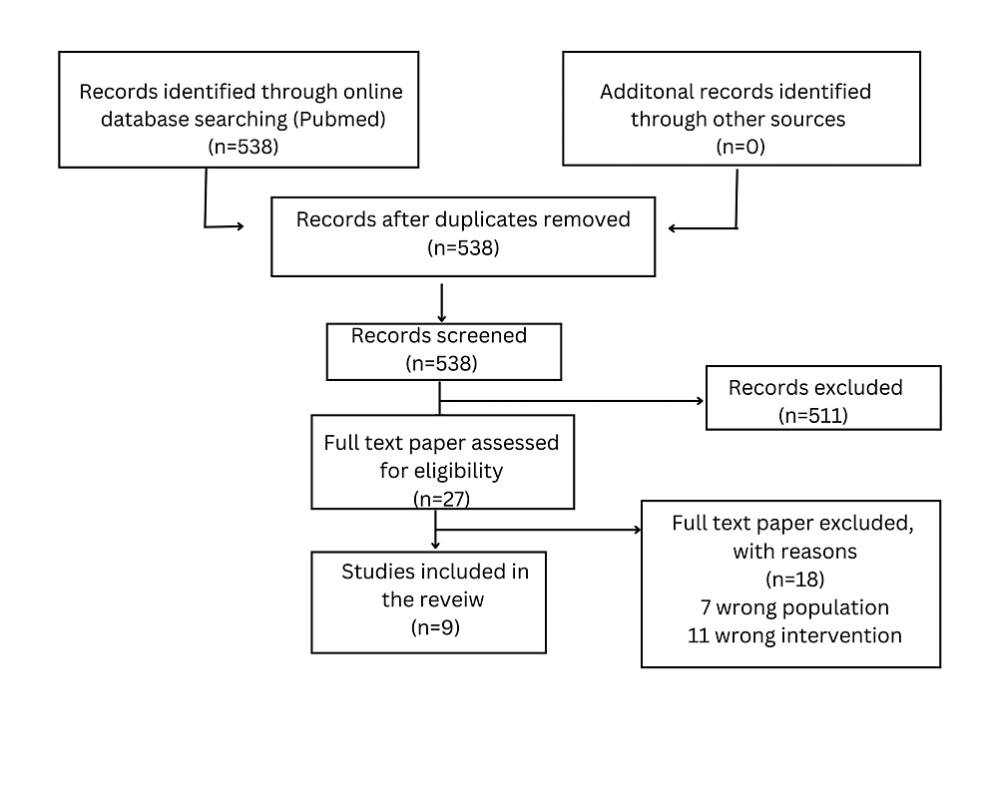
A flowchart presenting the literature searches and included studies Image created by the authors, Anushree Singh and Anuj R Varma.

This review pivots to analyzing the response of whole-body vibration therapy given to osteoporotic elders and postmenopausal women. Online databases such as PubMed and Google Scholar were thoroughly searched for potentially relevant studies. For panoramic analysis, the search strategy was tailored to individual databases, and the following keywords were used: vibration therapy, whole-body vibration therapy, vibration training, vibrotherapy, biomechanical stimulation, mechano-stimulation, osteoporosis, senile osteoporosis, and postmenopausal osteoporosis. Clinical trials and randomized controlled trials with publication dates within the last 10 years were included in this review article. The article search was confined to trials on osteoporotic elders and postmenopausal women. Trials involving pregnant or perimenopausal women and adolescents were excluded. Clinical trials where vibration therapy was used as a secondary treatment modality, like post-fracture repair surgery, were excluded. All references targeted the effects of whole-body vibration therapy on bone mineral density, muscle strength, balance, and postural control. In total, nine clinical trials were included in the final review. The selection of the included studies is illustrated in the flowchart below (Figure 1).

Osteoporosis

**Figure 2:**
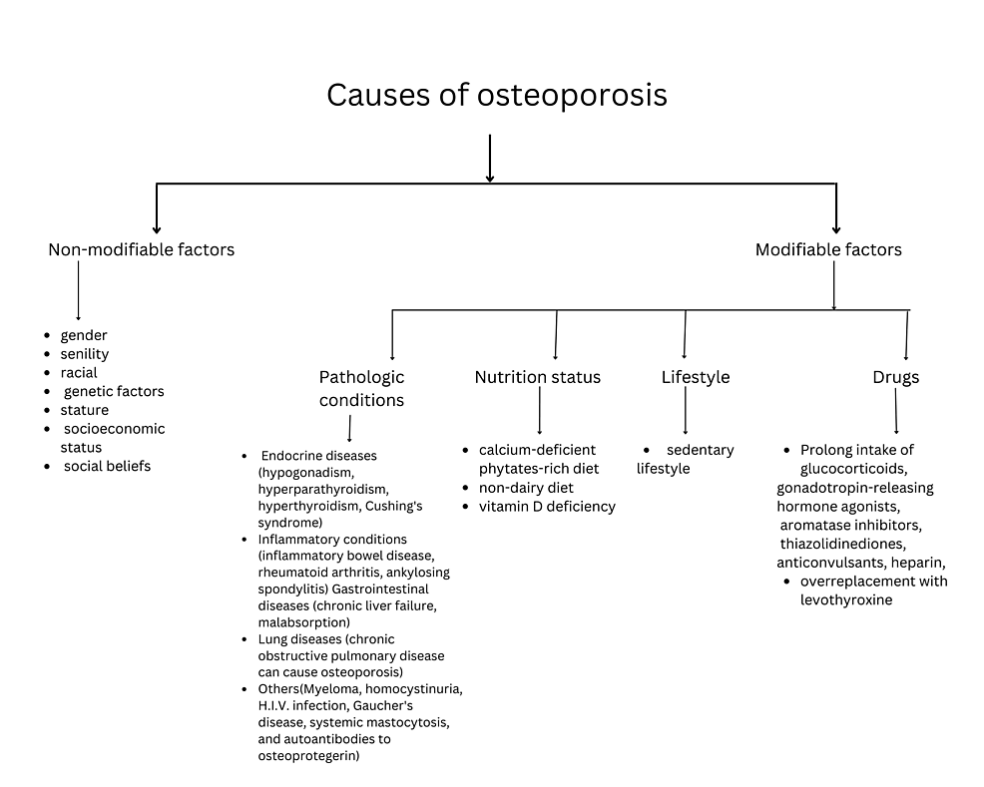
Causes of osteoporosis Image created by the authors, Anushree Singh and Anuj R Varma.

Osteoporosis can be described as a reduction in bone density that causes microarchitectural deterioration of the bone and predisposes to an increased chance of fracture even with minor insults to the bone [4]. Especially in geriatric populations with an increased risk of falling, the likelihood of sustaining a fracture also increases [4]. The peak of bone mass is seen between 20 and 45 years of age, after which decline is usually recorded [4]. The fall is stark in women because of estrogen deficiency after menopause. Imbalances in the osteoblastic-osteoclastic cycle and differentiation of bone marrow stem cells into adipocytes can cause age-related bone loss [4]. Risk factors for osteoporosis can be described as non-modifiable and modifiable factors, as shown in Figure 2. Non-modifiable factors include gender, senility, and racial and genetic factors [4,5]. Prolonged exposure to estrogen [5,6], poor sun exposure, and a poor diet due to socioeconomic and sociocultural beliefs are predominantly some of the leading causes of the higher incidence of osteoporosis in women, especially in India [5]. Studies have shown a rising prevalence of osteoporosis in the geriatric population, with a marked increase in Indian women more than men [5,7-9]. Early menarche and early menopause in Indian women have been found to predispose them to a higher risk of having osteoporosis than other races like Caucasians [5,10,11]. In addition to this, Asian Indian women have been observed to have lower bone mass than black and Caucasian women [5,12,13]. Ethnic variations can be due to polymorphisms in the genes coding for vitamin D receptors [5,14]. A correlation between estrogen and vitamin D receptor polymorphism has been proven in various studies on postmenopausal women [5,15-18]. Another single gene disorder, the LRP5 mutation, exhibits a risk of developing osteoporosis [4,5].

Nutritional status, lifestyle, pathologic conditions, and drugs can contribute to modifiable risk factors for osteoporosis. India's calcium-deficient, phytate-rich diet is responsible for low bone mineral density [5,19]. The daily requirement of calcium (600 mg) is barely met by a non-dairy diet, thereby contributing to an increased risk of developing osteoporosis [5,20]. Even though vitamin D is produced in the body through sun exposure, its deficiency is still quite common. Inadequate intake, malabsorption, high levels of melanin in the skin, or traditional forms of clothing can be responsible for this [5,21].

Low body mass index, small stature, and low adipose tissue percentage are proportional to low bone mineral density [5, 22-24]. In women, a weight less than 60 kg and a height less than 155 cm have predisposed them to osteoporosis [5,25]. An urbanized lifestyle of fast-paced yet sedentary living leads to the deterioration of bone mineral density. A corporate lifestyle has not only decreased physical activity but also decreased sun exposure time, hence further contributing to vitamin D deficiency [5,21]. Endocrine diseases like hypogonadism, hyperparathyroidism, hyperthyroidism, and Cushing's syndrome; inflammatory conditions like inflammatory bowel disease, rheumatoid arthritis, and ankylosing spondylitis; gastrointestinal diseases like chronic liver failure and malabsorption; and lung diseases like chronic obstructive pulmonary disease can cause osteoporosis. Myeloma, homocystinuria, HIV infection, Gaucher's disease, systemic mastocytosis, and autoantibodies to osteoprotegerin may also lead to loss of bone mass [4,5]. Prolonged intake of glucocorticoids, gonadotropin-releasing hormone agonists, aromatase inhibitors, thiazolidinediones, anticonvulsants, heparin, and overreplacement with levothyroxine can cause osteoporotic changes in the bone tissue [4,5,21]. Heavy smoking and alcohol consumption higher than three units a day can also be considered risk factors for osteoporosis in women [4,5,25].

Senile Osteoporosis

Physiologically, bone formation (osteoblastic activity) and bone reabsorption (osteoclastic activity) happen in a balanced form. This balance depends on factors like age, endocrine function, nutrition, genetics, and the maintenance of bone mass and density [4]. The imbalance between these two processes produces various types of bone metabolic disorders. The most common type of disorder is osteoporosis, where we see a diffuse reduction in bone density due to loss of bone mass. It occurs because osteoclastic activity exceeds osteoblastic activity. It is associated with a normal bone mineral-to-matrix ratio. Factors like senility, post-treatment immobilization (like in a bedridden patient), post-menopause, protein deficiency (which may be due to inadequate intake or excessive excretion or malnutrition or malabsorption), endocrine disorders like Cushing's disease, hypothyroidism, and drug therapies like phenobarbitone therapy and long-term steroid therapies may manifest osteoporosis. Out of all these causes, senility in males and menopause in females are the most common [4,5].

Post-Menopausal and Other Types of Osteoporosis

After about five to 10 years of menopause, the supply of the precursors of estrogen reduces, and a woman enters the "true state of menopause," where a sharp decline in estrogen levels is seen. Estrogen plays a pivotal role in preventing osteoporosis through various mechanisms. It inhibits the release of interleukin-1 (IL-1), reduces osteoclastic activity, increases calcium absorption in the stomach, stimulates calcitonin secretion from the thyroid gland, and increases vitamin D, which leads to bone mineralization [4,5,26,27]. Post menopause, about 3% to 5% of the bone mass is lost per year. Generally, patients are asymptomatic unless fracture-like complications occur. The dorso-lumbar spine is the most frequent site of fracture seen in these cases [26]. Colles' fracture and fracture of the neck of the femur can also be associated with osteoporosis. A diminutive loss of height increases kyphosis due to compression of the anterior part of the vertebral bodies, and back pain is seen in patients. Such fractures produce a very high probability of mortality and morbidity in older women [4,5,26,27].

Other types of osteoporosis include idiopathic osteoporosis, which occurs without any underlying cause [4]. Secondary osteoporosis is associated with any pathological condition and drug therapy [4]. Glucocorticoid-induced osteoporosis occurs in patients with systemic inflammation and chronic lung diseases. If patients are given more than 7.5 mg of glucocorticoids for over three months, they are at higher risk [4]. Pregnancy-induced osteoporosis is rare; the patient presents in the second or third trimester with a vertebral fracture [4].

Investigations and Findings

An X-ray can show loss of bone mass (evident only after 30% of the original mass is already lost), the vertical height of vertebrae, a codfish-like appearance of the vertebral body (biconvex vertebrae), and the ground-glass appearance of bones. Singh's index, metacarpal index, and vertebral index are used to determine the grade of osteoporosis. Dual-energy X-ray absorptiometry (DEXA) is X-ray-based bone densitometry, which is taken as the gold standard for quantifying bone mass [26,27]. Indications for dual-energy X-ray absorptiometry are low-trauma fractures in a patient older than 50 years of age. The presence of clinical risk factors, a 10-year fracture risk of more than 10%, and patients on more than three months of high-dose glucocorticoid therapy are an indication for dual X-ray absorptiometry. Assessment of response to osteoporosis treatment and progression of osteopenia to osteoporosis is done with DEXA. Those aged less than 50 with major risk factors for osteoporosis should also be screened [4]. The bone mineral density test reports a T-score that can help determine the specific types of bone degenerative diseases. For normal bone mass density, the T-score is between +2.5 and -1.0; for osteopenia, it is between -1.0 and -2.5; for osteoporosis, it is less than or equal to -2.5; and for severe osteoporosis, it is more than 2.5 with one or more fractures [26,27]. The fracture risk assessment tool (FRAX) helps calculate a patient’s 10-year fracture risk probability. This scale considers 11 risk factors and femoral neck raw bone mass density. Neutron activation analysis, bone biopsy, and biochemistry, including total plasma proteins and plasma albumin, urinary calcium/creatinine, and hydroxyproline/creatinine ratio, can also be analyzed [26,27]. Screening of endocrine, inflammatory, gastrointestinal, and lung diseases and neoplasia should be considered to rule out possible predisposing etiologies [4].

Treatment Approaches

Almost all of these conditions do not have a definitive cure. These are managed and controlled only by long-term medication. The main aim of these management techniques is to improve health, avoid disability, and maintain independence so one can take care of themselves for as long as possible. Generally, the mode of treatment in such cases involves a medical and orthopedic approach. Medical treatment includes improved diets, calcium supplements, androgen therapy, estrogen therapy, vitamin D, fluoride, alendronate and calcitonin, teriparatide, and denosumab [26,27]. Postmenopausal osteoporosis can be treated with bisphosphonates (alendronic acid, risedronate, ibandronate, zoledronic acid), with monoclonal antibodies (denosumab), with calcium and vitamin D, or with parathyroid hormones (teriparatide/abaloparatide), with hormone replacement therapy with progesterone and estrogen, with a selective estrogen receptor modulator (raloxifene), or with tibolone, which has partial agonist activity at estrogen, progesterone, and androgen receptors [4]. Bisphosphonates, monoclonal antibodies, calcium and vitamin D supplements, and teriparatide are efficacious in male osteoporosis [4]. Drug choices are similar for treating glucocorticoid-induced osteoporosis as in male osteoporosis, except for monoclonal antibody therapy [4]. The orthopedic approach involves weight-bearing exercises and prophylactic bracing [4,5]. Medical management is depicted below in Table 1.

**Table 1:** Medical management of osteoporosis

Class of drugs	Post-menopausal osteoporosis	Glucocorticoid osteoporosis	Male osteoporosis
Bisphosphonate	Alendronic acid, risedronate, ibandronate, and zoledronic acid	Alendronic acid, risedronate, ibandronate, and zoledronic acid	Alendronic acid, risedronate, ibandronate, zoledronic acid
Monoclonal antibody	Denosumab	-	Denosumab
Calcium/Vitamin D	Calcium (500-1000 mg), vitamin D (400-800 IU)	Calcium (500-1000 mg), vitamin D (400-800 IU)	Calcium (500-1000 mg), vitamin D (400-800 IU)
Human parathyroid hormone fragment	Teriparatide	Teriparatide	Teriparatide
Parathyroid hormone-related protein fragment	Abaloparatide	-	-
Hormone replacement therapy	With cyclic progesterone and estrogen	-	-
Selective estrogen receptor modulator	Raloxifene	-	-
Partial agonist for progesterone, estrogen, and androgen	Tibolone	-	-

With new advances in medicine, newer modalities for the treatment of osteoporosis are being explored. Vibration therapy is gradually being regarded as an effective intervention in the management of physiological and pathological disorders such as osteoporosis and sarcopenia, given its promising potential for enhancing bone mass and density. The vibration safely delivers a mechanical stimulus to patients who are physically restricted and are unable to exercise and rebuild musculoskeletal strength [28]. Vibration therapy protocols encompass the use of a wide range of frequencies and durations of exposure. The frequencies range from 12 Hz to 90 Hz, whereas the exposure period may stretch from two to 22 months. The therapy was explored not only as a primary mode of treatment but also as an adjuvant therapy. Trials have been conducted in combination with various pharmacological therapies. Bone mass is analyzed during and after treatment induction using X-rays, dual-energy X-ray absorptiometry, and high-resolution peripheral quantitative computed tomography. Biomarkers like alkaline phosphatase, N-telopeptide, and 25-hydroxyvitamin D are analyzed to quantify the progress. Individuals under protocol sometimes require calcium and vitamin D supplementation.

Vibration therapy

History

Dr. Gustav Zander (1857) was among the originators of mechanotherapy. He understood the importance of mechanical therapy and regular muscle exertion [29]. He invented various devices that combined exercise with mechanical oscillations [29]. In 1895, Dr. John Harvey Kellogg hypothesized that vibration therapy could help improve circulation and relieve constipation. He invented the full-body vibration machine [30]. Later, Russia used this full-body vibration plate to rehabilitate injured athletes. This assisted their athletes in winning 43 gold medals in the 1960 Olympics [30]. In Germany around the same time, rhythmic neuromuscular stimulation was developed by Biermann while working on the effects of cyclic massage on trunk flexing [31]. In 1961, NASA encountered a problem faced by astronauts sent on long-period missions, who developed significant bone weakness and muscle atrophy due to the absence of gravity. Astronauts relied on extensive exercises that induced the resistance of their body weight on Earth. To achieve the ideal effect, spacecraft were fitted with avant-garde devices, and astronauts would spend at least two hours of their day exercising. All this helped with the problem of muscle atrophy but not with the bone mass loss issue. After this, NASA developed systems with minimal vibration conduction in these pre-existing devices, like the treadmill vibration isolation system and cycle-ergometer vibration isolation system [3]. Russian scientists began to study the mechanics of full-body vibration with cosmonauts who returned from space after their missions to overcome their bone mass loss. They too discovered astonishing effects in the rehabilitation of cosmonauts, where bone density improved and muscle strength increased [30]. In 1995, Dr. Valery Polakov spent 438 days in space using vibration technology and established a world record. Their astronauts were able to spend more than 420 days in space at a stretch. At the same time, American astronauts could only spend 120 days in space [30].

Rhythmic oscillation therapy has been tested extensively. The primary goal was to standardize a safe frequency range and duration. Tests on turkeys, sheep, and rats were conducted with increased frequency to a level where it became sufficient to lift a 40 kg man or crack the steel body of the device itself [3]. In 2003, the European Space Agency performed the first bedrest study using a vibration training device for humans in Berlin [32]. Later in 2006, the German Aerospace Agency demonstrated the feasibility of using the same lightweight vibration training device under microgravity conditions during several parabolic flight campaigns. Its effect and influence were studied until 2010 [32].

Mechanism of Action

**Figure 3:**
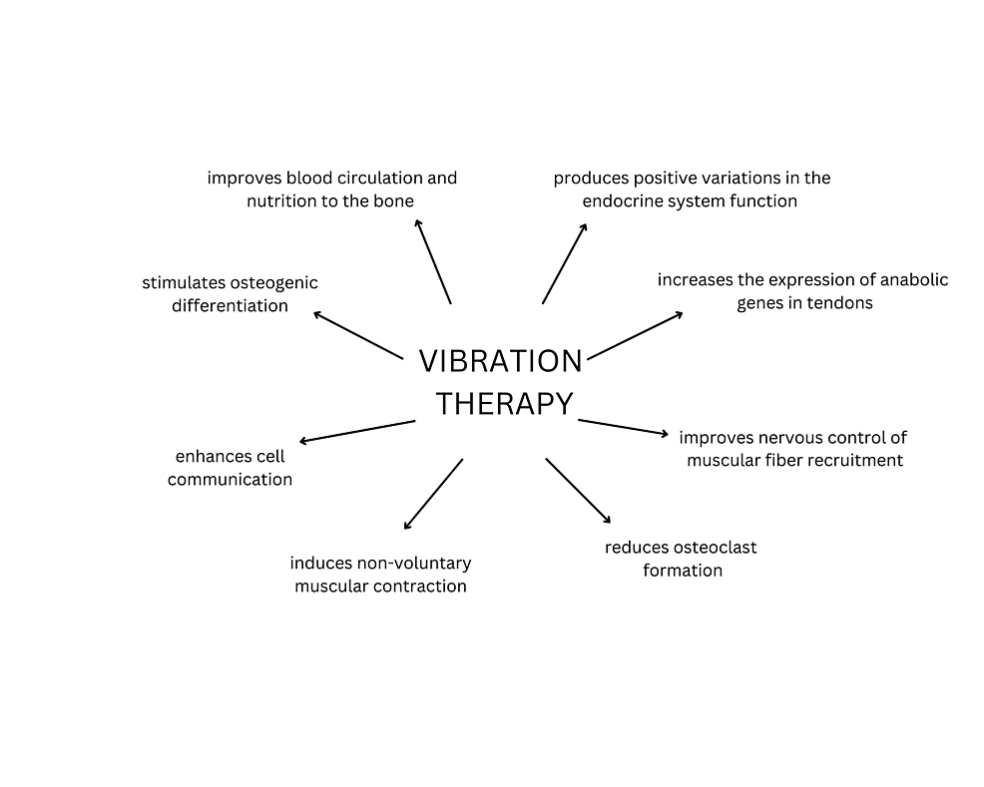
Mechanism of vibration therapy Image created by the authors, Anushree Singh and Anuj R Varma.

Whole-body vibration therapy affects bone metabolism, muscle function, muscle training, and the endocrine system, as shown in Figure 3. Vibration therapy provides anabolic mechanical signals to the bone and musculotendinous systems [28]. It improves blood circulation to the bones, ensuring an improved nutrition supply. Human adipose-derived stem cell differentiation into osteoblasts is facilitated by vibration therapy [28]. It reduces the expression of the dendritic cell-specific transmembrane protein receptor and receptor activator of nuclear factor kappa beta in osteocytes, thereby inhibiting excessive osteoclast formation [28]. It improves bone health by amplifying gap junctional communication in osteocytes [28]. Vibrations activate the tonic vibration reflex and induce non-voluntary muscular contraction [28]. Due to the excitation of IA afferent signals from the neuromuscular spindle, activation of the proprioceptive sensory system occurs. These signals then activate the alpha-motoneurons, leading to the involvement of previously inactive muscle fibers [28]. Golgi tendinous organs (GTO) are sensitive to variations in tension and may also get affected [28]. Besides the musculoskeletal system, the whole-body application of vibration therapy enhances the endocrine system's functioning. Growth hormone increases after positive feedback is induced by artificial gravity from the vibration therapy machine [28].

Cabello et al. conducted a randomized clinical trial to determine the efficacy of short-term full-body vibration therapy on bone mass and structure in the geriatric population. The trial included 49 volunteers divided into two groups randomly. One group was taken as a control, and another group of 24 elderly people was given full-body vibration therapy. The volunteers were trained on a vibrating platform in a squatting position three times a week. The trial lasted for 11 weeks. The outcomes were quantified in the pre-and post-phase therapy using dual-energy X-ray absorptiometry and peripheral quantitative CT scanning. Cabello et al. used analysis of covariance to compare the control and the exposed groups, and to compare within the same group, they used analysis of variance. All the volunteers were analyzed for the covariates: age, height, subtotal lean mass, and daily calcium intake. The study did not observe any bone mineral content and density changes by the end of 11 weeks. They concluded that the therapy was insufficient to produce changes in bone mineral content and density [33].

Another study by Shanb et al. attempted to analyze the effect of whole-body vibration versus magnetic therapy in conjunction with a standard medical regimen. The regimen included alendronate sodium, calcium, and vitamin D. The study had 85 participants (between 65 and 70 years of age) randomly allocated into three groups. The first group had 30 participants who were given whole-body vibration therapy for 16 weeks. Each session was for 25 minutes and was repeated twice weekly. The second group also had 30 participants who were given magnetic therapy for 50 minutes twice weekly for 16 weeks. The third group had 25 participants who were given alendronate sodium, calcium, and vitamin D only. They took the lumbar spine and head of the femur bone density as a measure of the outcome. Dual-energy X-ray absorptiometry was used for determining the density of bone, whereas venous blood samples were for serum levels of calcium and vitamin D. After the analysis of variance, the study concluded that there was a significant increase in bone mass density in both whole-body vibration therapy and magnetic therapy, along with the pharmacological regime. Only the group on the standard regimen failed to show improvement in bone density. Shanb et al. concluded that magnetic therapy and whole-body vibration therapy were equally effective in increasing bone mass density and could potentially be used for treating osteoporosis along with the standard pharmacological regime [34].

In December 2013, Clinical Interventions of Aging published a randomized clinical trial by Lai et al. that studied 28 postmenopausal women for six months for the impact of large magnitude and frequency vibration therapy on their lumbar spine’s bone density. They were allotted to either the treatment group or the control group. The treatment group received a high frequency of 30 Hz and a high magnitude of 3.2 g of vibration therapy in a standing position for five minutes, three times a week. The findings were investigated using dual-energy X-ray absorptiometry. The bone mass density of the treatment group increased by 2.032%, whereas the control group decreased by 0.046%. Lai et al. concluded that six months of high frequency and magnitude vibration therapy effectively boosted the lumbar spine’s mineral density in osteoporotic women and could potentially be an alternative intervention for treatment [35].

The same year, Stolzenberg et al. did a randomized controlled trial, which was published in the Journal of Musculoskeletal Neuronal Interact. It was done in pursuit of findings on the effect on bone density of the lower leg after nine months of resistance exercises combined with balance training or whole-body vibration therapy in osteoporotic postmenopausal women. The trials started with 68 volunteers, who were distributed into two groups. One group was given balance training and resistive exercises, and another was given vibration therapy. Out of the 68 volunteers, 57 completed the study. The therapy was induced twice daily for nine months. Then they were evaluated using peripheral quantitative computed tomography of limb bones that included the ulna, tibia, radius, and fibula, especially at the epiphysis and diaphysis. Stolzenberg et al. found a significant improvement in bone mineral density and strength at these sites and that both of the interventions (vibration training and balance training) had almost no difference in the outcomes of the two groups. They concluded that the resistance exercise program with either training technique effectively improved the density of the bones in the volunteers [36].

In 2015, Brunetti et al. conducted a randomized trial that evaluated the effect of localized repetitive vibratory stimuli on the quadriceps of the voluntarily extended knee on leg power, balance, and bone density in women post-menopause with osteoporotic changes. It was a double-blinded control study with 40 volunteers who were divided into two groups, namely the control and treatment groups. The therapy consisted of three applications of 100 Hz and 300-500 mm per day for 10 minutes for three consecutive days. The proximal femur’s T-score was analyzed 14 days preceding and 360 days post-therapy. The leg power and body balance were evaluated one day prior, 30 days, and 360 days after the intervention. Brunetti et al. observed no apparent improvement in the T-Score of the treatment group compared to the pre-treatment and post-treatment phases. However, in the control group, the T-score significantly decreased throughout the period of the study. They also recorded improved balance and explosive leg power in the treatment group on the 30th and 360th days post-therapy. They concluded that short-term repeated vibratory stimuli applications were non-invasive, safe, and sufficient enough to considerably curtail the progression of demineralization of bones in women post-menopause [37].

A clinical trial by Jepsen et al., in 2019, studied the cumulative effect of parathyroid hormone therapy with whole-body vibration exercises as a treatment for postmenopausal osteoporosis. The trial started with 35 women, who were divided into two groups randomly. The first group was given teriparatide (20 mg per day) and three sessions of 12-minute vibration exercise weekly. The women in the second group were asked to take only 20 mg of teriparatide daily. After 12 months, they were tested for bone mineral density and microarchitecture with markers and the circular routine. The data received was compared with the data recorded before the intervention and analyzed using robust cluster regression for a treatment analysis. Jepsen et al. observed that both groups had substantial gains in bone density. The lumbar spine’s density showed stark improvement. On the contrary, hip joint density did not show any changes post-therapy. Other parameters also increased compared to pre-interventional values but did not differ between the two groups. They concluded the article with an inference that the combination is therapeutically effective in treating postmenopausal osteoporosis [38].

An open randomized trial was performed by Wadworth et al. in 2020 to find the repercussions of whole-body vibration therapy on the bodily function of the elderly. Volunteers were randomly segregated into two groups, where one group was given controlled simulated vibration therapy and the other group was assigned whole-body vibration therapy. The participants were exposed to vibrations three times a week for 16 weeks. Treatment was initiated with 5x1-minute bouts of 6 Hz per 2 mm and slowly increased to 10x1 minutes at 26 Hz per 4 mm. Volunteers were asked to keep their knees flexed throughout the procedure. At the same time, the other group performed exercises that simulated vibrational therapy. By the end of eight and 16 weeks of therapy and three, six, and 12 months post-therapy, an assessment for physical function was done using a parallel walk, a 10 m timed walk, and a timed up and go test, along with the Barthel Index questionnaire. Wadworth et al. concluded that physical function significantly improved in the group exposed to the therapy. The exposed participants showed positive results after six months in the 10 m timed walk test and 12 months in the parallel walk. The study also reported high compliance and almost no side effects from the therapy [39].

Similarly, another clinical trial done by Eldeeb et al. attempted to analyze the impact of vibration therapy on the musculature and density of the femur bone and lumbar vertebra in postmenopausal women. The study started with 43 participants who had low bone mineral density. They were blindly distributed into two groups: one control group and another that received the therapy twice a week for 182 days. In addition to the therapy, both groups were given vitamin D and calcium daily. Eldeeb et al. used DEXA to evaluate bone mineral density in the femur and lumbar vertebrae for pre- and post-interventional analysis. For muscle work analysis, they used the Qualisys gait analysis system. It included analyses of hip power absorption and generation by the joint’s flexors and extensors, knee power absorption by the hamstring, generation by the quadriceps, and absorption by the quadriceps during the pre-swing and loading response, ankle power absorption by dorsiflexion and plantar flexors, and ankle power generation from plantar flexors. Eldeeb et al. observed a notable increase in the muscle work in the hip, knee, and ankle in addition to an improvement in the density of the femur and lumbar vertebrae after the therapy in the experimental group [40].

Kienberger et al. did a randomized controlled trial on 65 post-menopausal women to evaluate the effects of whole-body vibration on bone mineral density, muscle strength, and postural control. Participants were divided into three groups, namely the vibration training group, the resistance training group, and the control group. The vibration training group was trained twice a week for 12 months using vibrations of 18 Hz and an amplitude of 2 mm in different knee joint positions. Bone mineral density was then analyzed on day 0 and after 12 months of intervention. Resistance training started with a warm-up on a cycle followed by balance exercises. Later, the training sessions incorporated six different resistance training machines: leg flexion, leg extension, leg press, leg abduction, latissimus machine, and pulley. Each session lasted 45 minutes and was held twice a week for 12 months. The control group was asked to continue with the routine activity and not engage in any new form of physical training. The DEXA was used to evaluate bone mineral density on day 0, in the sixth month, and in the 12th month of intervention. A Gleichgewichts test for postural control and an isokinetic muscle strength assessment was used to assess the muscle strength in the participants on day 0, in the sixth, 12th, and 15th months from the start of the trial. They observed that, compared to the control group, neither the vibration training group nor the resistance training group demonstrated appreciable changes in bone mineral density T-score values after the intervention period. All training groups saw a significant increase in isokinetic strength, except the flexors of those in the vibration training group. They concluded that there were no significant changes due to the training and that all three groups could maintain their bone mineral density equally. Only resistance training provided improved posture control for the participants [41].

Out of nine articles, three were based on geriatric populations. Two of these articles concluded with a positive outcome after vibration therapy. The other six articles targeted the effects of vibration therapy on postmenopausal women. Five of these articles favor vibration therapy as being effective as a treatment modality for osteoporosis and preventing progressive degeneration of bone mineral density. Two of these five articles proved that vibration therapy could be combined with a pharmacological regimen of vitamin D and calcium supplements or parathyroid hormone for better results. There are three stated improvements in bone mineral density with vibration therapy as a primary intervention. One of the articles also states that the effect of vibration therapy is equivalent to the impact of magnetic training for the treatment of osteoporosis. One article found no difference in the bone mineral density of the trial and control groups. All inferences have been summarized in Table 2 below.

**Table 2:** Summary of reviewed articles DEXA: dual-energy X-ray absorptiometry; pQCT: peripheral quantitative computed tomography; WBV: whole-body vibration group; SIM: simulated whole-body vibration group; CON: control group; BMD: bone mineral density; VIB: whole-body vibration exercise group; BAL: balance training group; VC: vibrated and contracted group; NV: non-vibrated group; HR-pQCT: high-resolution peripheral quantitative computed tomography; VT: vibration training group; RT: resistance training group; CG: control group

Article/Study	Sample size	Intervention	Frequency and amplitude	Assessment parameter	Result
Cabello et al. [33]	49 participants (n = 49) were divided into two groups: the whole-body vibration group (n = 24, age=75.2 ± 4.7 years) and the control group (n=25, age=74.8± 4.9 years).	The vibration group was trained with the knee slightly flexed in an erect posture with feet apart on the platform three times weekly for eleven weeks, and group 2 was taken as the control.	40 Hz and 2 mm	DEXA and pQCT	No changes in bone mineral content and density. Moreover, at the tibia, total, trabecular, and cortical volumetric bone mineral density decreased (all p<0.05).
Shanb et al. [34]	85 participants (n=85), divided into three groups, group I (n=30, age=64.1±4.4 years), group II (n=30, age=63.9 ± 3.9 years), and group III (n=25, age 64.50±4.03 years)	Group 1 was exposed to two sessions of vibration therapy for 25 minutes weekly for four months along with standard pharmacological treatment; group 2 was trained with magnetic therapy for 50 minutes twice weekly for four months along with standard pharmacological treatment; and group 3 was kept on pharmacological treatment alone.	Vibration therapy: 20–50 Hz, 1-3.4 mm Magnetic therapy: up to 100 Hz and 85 gauss of intensity	T-score	Significant increases in bone mineral density of the femoral head and lumbar spine were noted in groups 1 and 2 in comparison to group 3 (both had p<0.05). They observed no differences in the outcomes of these two interventions.
Lai et al. [35]	28 postmenopausal women (n=28) were divided into the WBV group (n=14, age=6-.1±7.1 years) and CON group (n=14, age=62.4±7.1 years)	The WBV group was given vibration therapy in a standing position for five minutes, three times weekly, for six months.	30 Hz; magnitude: 3.2 g	BMD	BMD in the trial group increased by 2.032% (p=0.047), while it decreased in the control group by 0.046%.
Stolzenberg et al. [36]	57 postmenopausal women (n=57) were divided into the VIB group (n=34, age=67.2±3.7 years) and the BAL group (n=34, age=66.0±4.5 years).	Both groups were trained twice weekly for nine months, along with resistance exercises.	VIB: Standing: 22 Hz (3.9 g)–24 Hz (9.3 g); squatting and standing: 22 Hz (3.9 g)–24 Hz (9.3 g); and keen flexion: 2 Hz (0.6 Hz). BAL: A progressive proprioceptive and balance program was used.	pQCT	Significant improvement in the bone density and strength of the tibia, fibula, radius, and ulna. Although there was no significant difference in the responses to the two pieces of training.
Brunetti et al. [37]	40 postmenopausal women (n=40), divide into two groups, the VC group, and the NV group.	The VC group was given three sessions per day, each lasting ten minutes, for three days.	100 Hz, 300-500 μm	T-score	The T-score of the VC group did not change in respect of the baseline values in one year (pretreatment: -2.61±0.11, post-treatment: -2.62 ± 0.13), but in NV subjects it decreased significantly from -2.64 ± 0.15 SD down to -2.99 ± 0.28 SD.
Jepsen et al. [38]	35 postmenopausal women (n=35), divided into two groups, WBV+Teriparatide group (n=17, age=69.5 years) and only Teriparatide (n=18, age=69.9 years)	The WBV+ teriparatide group was trained at home for twelve minutes twice weekly for twelve months along with oral teriparatide every day whereas the teriparatide group was kept only on oral teriparatide for twelve months.	30 Hz, 1 mm	DEXA, HR-pQCT, and bone turnover markers	BMD of the lumbar spine in the WBV + teriparatide group increased by (mean ± SD) 8.90% ± 5.47 and the teriparatide group by 6.65% ± 5.51. The effect of adding WBV to teriparatide showed a statistically significant increase in BMD, 2.95% [95% CI = 0.14–5.77; p = 0.040].
Wadsworth et al. [39]	117 participants (n=117), divided into three groups: the WBV group (n=36, age=79.4±1.1 years), SIM group (n=35, age=83.7±1.2 years), and the control group (n=46, age=84.3±1.3 years).	The WBV group was given vibration therapy, and the SIM group was given a simulation of the therapy, both three times weekly for 16 weeks in isometric knee flexion.	From 6 Hz/2.0 mm to 26 Hz/4.0 mm	Timed Up and Go test Parallel Walk test 10 m timed walk test.	Whole-body vibration therapy produced clinically relevant therapeutic effects in the trial group.
Eldeeb et al. [40]	43 postmenopausal women (n=43) were divided into the WBV group (n=22, age=55.09±4.19 years) and control group (n=21, age=57.29 ± 4.44 years).	The WBV group was given calcium and vitamin D supplements along with whole-body vibration exercises twice weekly for 24 months, and the other group was taken as the control and was given only medications.	20 to 35 Hz, 2.5 to 5 mm, calcium (1200 mg), and vitamin D (800 IU) once daily.	BMD, Qualisys gait analysis system, and DEXA	There was a significant increase in hip muscle work, knee muscle work, ankle muscle work during gait, and BMD of the femur and lumbar vertebrae in the WBV group.
Kienberger et al. [41]	61 postmenopausal women (n=61) divided into three groups VT (n=18, age=56.1±5.1 years), RT (n=22, age=62.8±6.8 years), and CG (n=21, age=58.7±8.2 years)	Both VT and RT were trained twice weekly for 12 months. The CG was asked to continue with their day-to-day activities.	18 Hz, 2 mm	DEXA, Gleichgewichts test, and isokinetic muscle strength assessment	There was no significant variation in the parameters post-intervention. All three groups maintained their BMD.

## Conclusions

It can be concluded that vibration therapy showed positive outcomes as a treatment modality to improve bone mass density and postural control in postmenopausal women and geriatric populations. However, the independence of the therapy should be explored more extensively to determine whether it is sufficient to be used alone or if it must be combined with medical treatment for the best results. Vibration therapy also shows promising results in regaining muscle mass and function after degeneration. It reinforces the blood supply to the bones and reduces osteoclast formation. It reactivates the inactive muscle fibers and the neuronal and proprioceptive sensory systems around them. Vibration therapy can be regarded as an alternative to stimulating physically restricted patients with mechanical stimulation to rebuild musculoskeletal strength. It is safe, non-invasive, easy to use, and an effective treatment option that still requires more research to explore and extend its potential. More extensive studies are needed to analyze the adequate amplitude, frequency, and duration of the therapy. In addition, it can also be concluded that regular physical activity, adequate nutrition, and early diagnosis can immensely contribute to a reduction in the incidence of age-related bone degenerative disorders.
